# Dissecting antimicrobial logic: species-specific strategies for intracellular pathogen defense

**DOI:** 10.1128/msphere.00091-25

**Published:** 2025-09-10

**Authors:** Bryan D. Bryson

**Affiliations:** 1 Department of Biological Engineering, Massachusetts Institute of Technology 2167, Cambridge, Massachusetts, USA; 2 Ragon Institute of Mass General, MIT, and Harvard 200750 , Cambridge, Massachusetts, USA; Virginia-Maryland College of Veterinary Medicine, Blacksburg, Virginia, USA

**Keywords:** innate immunity, host-pathogen interactions, phagosome

## Abstract

Control of intracellular pathogens is a critical element of host defense. Defining the molecular mechanisms by which the host restricts or eliminates these pathogens may inform the development of novel immunotherapeutics and antimicrobial strategies, particularly in the face of rising antibiotic resistance. In parallel, understanding how pathogens subvert these immune responses may yield new approaches to disrupt virulence rather than viability. Yet, the precise mechanisms by which primates—and especially humans—achieve intracellular pathogen control remain poorly understood. Five years ago, I reflected on the complexity of interferon-induced control of *Legionella pneumophila* in a murine infection model. In this review, I revisit those questions considering emerging evidence, highlighting how cross-species comparisons and context-specific immune programs are reshaping our understanding of host-pathogen interactions and the logic of antimicrobial defense.

## INTRODUCTION

In prior work, Price and colleagues built upon a previous study by Pilla et al., who generated mice deficient in *Nos2, Cybb, Irgm1,* and *Irgm3*, all genes induced by IFN-γ and implicated in pathogen defense (1, 2). Surprisingly, despite the deletion of four genes previously implicated in intracellular pathogen control following interferon stimulation, macrophages lacking these were still able to restrict *L. pneumophila* replication when stimulated with IFN-γ (2). Price and colleagues built upon this previous study by interrogating the contribution of two additional genes, *Casp4* (encoding caspase 11 which triggers pyroptosis) and *Acod1* (encoding immune-responsive gene 1 which produces itaconate) (3–5). These two genes were the subject of additional studies exploring the role of candidate antimicrobial genes in control of *L. pneumophila*. In the Price study, macrophages lacking any one of these six genes still restricted *Legionella* replication. Only when all six genes were knocked out was IFN-γ-mediated control fully lost. This highlights a model of distributed antimicrobial control, rather than one dependent on a single critical gene.

What distinguishes the Price study is the demonstration that redundant and overlapping functions among ISGs collectively sustain control of *Legionella*. This contrasts with earlier models in which single-gene knockouts (e.g., *Nos2*, *Irgm1*) resulted in a collapse of antimicrobial control. For example, prior work by Macmicking demonstrated that loss of NOS2 or Irgm1 abrogated much of the antimicrobial control of *Mycobacterium tuberculosis* (Mtb) induced by IFN-γ treatment (6). Matta and colleagues demonstrated that RNF213 is required for the enhanced control of *Toxoplasma gondii* upon IFN-γ treatment; however, others have shown other genes such as guanylate binding proteins (GBPs) to be important for IFN-γ-mediated control (7–12).

Collectively, these findings raise key questions about the logic of IFN-γ-induced pathogen control. The Price study suggests that *L. pneumophila* clearance relies on a constellation of ISGs acting in parallel. If *Legionella* control followed a linear six-gene pathway, disrupting any one gene should impair defense. Yet, this is not what Price et al. observed—supporting a distributed, non-hierarchical model. While multiple ISGs have been implicated in control of other pathogens, how their combinatorial disruption affects pathogen control remains largely unexplored. Testing whether the same six genes contribute to IFN-γ-mediated control of other pathogens—such as Mtb—would validate the generalizability of this distributed model. For example, while a previous study in Mtb knocked out Irgm1 or NOS2 individually, might their combined disruption more completely abrogate IFN-γ-mediated control (6)? For these reasons and more, the Price study highlights opportunities to re-evaluate previously implicated genes for their joint roles in pathogen control.

The model proposed by Price and colleagues compels us to revisit fundamental assumptions about antimicrobial gene function. It prompts key questions: How do macrophages compensate when individual ISGs are lost? Are distinct pathogens vulnerable to unique combinations of antimicrobial pressures? And critically, can we translate this combinatorial model of control across species? Given that many of the genes implicated by Price show species-specific expression and regulation patterns, how might we integrate evolutionary adaptation into this multi-gene model for pathogen control?

In this review, I explore how comparative insights into antimicrobial control across species can illuminate principles of context-specific immunity—and ultimately inform new strategies to enhance pathogen clearance in humans.

## NITRIC OXIDE AND THE CONTEXT-SPECIFIC LOGIC OF ANTIMICROBIAL METABOLISM

Several antimicrobial metabolites have been identified, and their activity against intracellular pathogens has been characterized using a combination of genetic approaches and axenic culture experiments. Antimicrobial metabolites include reactive oxygen and nitrogen species, itaconate, and hypochlorous acid (13–15). Among the diverse antimicrobial metabolites produced by immune cells, reactive nitrogen species (RNS)—particularly nitric oxide (NO)—have played a central role in models of intracellular pathogen control (16). In mice, NO production is driven by the enzyme inducible nitric oxide synthase (iNOS, encoded by *Nos2*), which is robustly induced by IFN-γ in murine macrophages and has been shown to restrict replication of pathogens including *Mycobacterium tuberculosis*, *Listeria monocytogenes*, and *Leishmania major* (6, 17–19).

In contrast, the role of nitric oxide in human innate immunity remains unresolved (14, 16, 20–22). Many papers report that human macrophages produce minimal NO *in vitro*, even when exposed to classical activators such as IFN-γ and LPS, as measured by the Griess assay or other standard approaches (23). This has led to the suggestion that iNOS is not a functional part of the human macrophage antimicrobial repertoire. Others, however, have reported iNOS protein in human tissue samples—including inflammatory lesions or granulomas—suggesting that expression may occur under specific *in vivo* conditions that are not recapitulated in standard culture systems (24). Recent single-cell and spatial transcriptomic studies have rarely detected NOS2 expression in human myeloid cells, reinforcing the idea that if iNOS is used by human macrophages, its expression is highly context-dependent (25, 26).

Mechanistic studies suggest that this difference is not merely environmental but may reflect deep regulatory divergence between species (22, 23). A chromatin-based model of *NOS2* regulation has emerged, supported by data showing that the human *NOS2* locus contains a 75 kb insertion downstream of the gene and is organized across two topologically associated domains (TADs)—unlike the single TAD structure in mice (23). Moreover, key enhancer elements driving IFN-γ responsiveness in murine macrophages are absent or non-conserved in humans (27).

From the perspective of context-specific immunity, these findings raise important conceptual questions. Why do mice and humans differ so starkly in their reliance on nitric oxide as an antimicrobial strategy? Rather than viewing this as a technical discrepancy or evolutionary flaw, it may reflect species-specific optimization of immune metabolism. Mice may rely more heavily on NO as a frontline effector, while humans may favor alternative pathways. This divergence underscores the importance of interpreting host-pathogen interactions in their species- and context-specific immunological landscapes, rather than assuming a conserved set of effectors across mammals.

## ITACONATE AS A CONTEXTUALLY TUNED ANTIMICROBIAL METABOLITE

Itaconate, generated by the decarboxylation of cis-aconitate by the mitochondrial enzyme aconitate decarboxylase 1 (ACOD1, encoded by *IRG1*), has emerged as a key immunometabolite with dual roles in host defense and immune regulation (28–30). In mice, itaconate is robustly induced in macrophages following IFN-γ or TLR stimulation and has been shown to inhibit the growth of a variety of intracellular pathogens—including *Mycobacterium tuberculosis*, *Salmonella enterica*, and *Legionella pneumophila*—both *in vitro* and *in vivo* (5, 31–36). Mechanistically, itaconate functions by targeting microbial metabolism (e.g., inhibition of isocitrate lyase) and by modulating host inflammatory responses through inhibition of succinate dehydrogenase and activation of Nrf2 signaling (37–40).

To causally demonstrate its role in antimicrobial defense, multiple studies have employed both genetic ablation of *Acod1* in mice and exogenous addition of itaconate derivatives (e.g., dimethyl-itaconate, 4-octyl itaconate) to infected cells or axenic cultures (31, 35). These studies converge on a model in which itaconate contributes to a layered antimicrobial program, often acting in synergy with other stressors such as reactive nitrogen species (35).

However, recent comparative genomic and biochemical studies reveal that humans carry a unique, apparently private mutation in *IRG1* that markedly reduces the catalytic efficiency of the enzyme, resulting in orders-of-magnitude lower itaconate production relative to mice and other mammals, including non-human primates (41, 42). This finding is striking: no other sequenced vertebrate appears to share this mutation, suggesting that the downregulation of itaconate synthesis may be a human-specific evolutionary event.

The implications of this divergence are substantial. Many functional studies of itaconate use doses that reflect murine or supraphysiologic concentrations, raising questions about their relevance to human immunity (31, 35, 38). For example, recent work by Chen and colleagues demonstrated that Salmonella Typhi growth in axenic culture could be inhibited by rodent-range itaconate concentrations—but not by concentrations likely achievable by human macrophages (35). Notably, this antimicrobial effect was amplified when nitric oxide donors were added, consistent with the idea that combinatorial stress from multiple antimicrobial metabolites is needed for effective pathogen control—echoing the model proposed by Price and Vance.

Interestingly, rare human IRG1 variants that enhance enzymatic activity have been identified though their frequency is low (41). Whether individuals harboring such variants exhibit enhanced control of intracellular pathogens, or differential inflammatory phenotypes, remains unknown. This opens the door to testable hypotheses: for instance, can human macrophages engineered to express high-activity IRG1 variants better control Mtb or Salmonella infection *ex vivo*? Could these variants influence infection outcomes *in vivo*?

From a context-specific immunity perspective, the itaconate story illustrates how metabolic arms of innate immunity are flexibly deployed across species. Mice appear to invest heavily in itaconate as a front-line antimicrobial metabolite, whereas humans may instead rely on other pathways. Importantly, unlike the regulatory complexity of NOS2, the species differences in IRG1 are largely encoded in the protein sequence itself, making them more experimentally tractable via targeted mutagenesis or allele swapping.

In this light, the human-specific attenuation of IRG1 function may not reflect a deficiency *per se*, but a recalibration of the immune metabolic repertoire—one that trades high-output itaconate synthesis for alternative, perhaps less energetically costly or more tightly regulated modes of pathogen control.

## REACTIVE OXYGEN SPECIES: A CONSERVED BUT CONTEXTUALLY VARIABLE DEFENSE

Reactive oxygen species (ROS) are among the most evolutionarily conserved antimicrobial mechanisms, produced primarily by the phagocyte NADPH oxidase (NOX2) complex upon pathogen recognition and phagocytosis. In both mice and humans, NOX2 activation generates superoxide radicals and downstream ROS species that contribute to microbial killing, often in synergy with phagosomal acidification and metal ion flux (43–46).

Unlike nitric oxide or itaconate, which show substantial species divergence in their production and function, the core ROS machinery is more broadly conserved. Mutations in components of the NADPH oxidase complex—such as *CYBB*, encoding the catalytic subunit gp91^phox^—cause chronic granulomatous disease (CGD) in both humans and mice, leading to recurrent infections with catalase-positive bacteria and fungi (47, 47, 48). This phenotypic convergence across species suggests that ROS is a functionally critical and non-redundant antimicrobial mechanism in many contexts.

However, the regulation and magnitude of ROS production may still differ between species and cellular contexts. A comparative transcriptomic study showed that following LPS stimulation, murine macrophages more robustly upregulate *Cybb* expression relative to human monocyte-derived macrophages or tissue-resident populations (49). While this suggests differential tuning of the NOX2 complex across species, the consequences for actual ROS output remain poorly characterized, particularly in controlled head-to-head functional assays.

Moreover, the contribution of ROS to pathogen control appears to be context-dependent even within species. For example, certain intracellular pathogens—such as *Salmonella*, *Mtb*, or *Leishmania*—actively remodel phagosomal compartments or express ROS-detoxifying enzymes, reducing their susceptibility to oxidative stress. In these cases, ROS may function more as a priming or signaling cue—triggering further immune responses such as inflammasome activation—than as a direct bactericidal agent (44, 50–52).

From a context-specific immunity perspective, this highlights a crucial distinction: ROS may be a conserved tool in the immune arsenal, but its deployment and effectiveness vary widely by pathogen type, tissue niche, and host species. While murine models have provided invaluable insight into NADPH oxidase biology, a deeper understanding of how ROS is modulated in human myeloid cells—and how its role differs from or complements other antimicrobial pathways—is still needed. The apparent conservation of function, juxtaposed with variation in inducibility and cellular deployment, makes ROS an informative case study in the spectrum between conserved effectors and context-specific logic. Thus, while ROS remains a broadly conserved mechanism, its deployment reflects nuanced immunological tuning rather than a fixed output.

## IRG PROTEINS AND THE EVOLUTIONARY REWIRING OF GTPase-MEDIATED IMMUNITY

Immunity-related GTPases (IRGs) are a family of interferon-inducible dynamin-like proteins that play central roles in host defense, particularly against intracellular pathogens. In mice, IRGs are highly expanded, with over 20 family members, including Irgm1, Irgm3, and other effector IRGs that target pathogen-containing vacuoles or cytosolic bacteria (53). This expansion allows for redundancy, functional specialization, and regulatory layering across different infection contexts.

In humans, by contrast, the IRG family is strikingly contracted. Only two genes are retained: IRGM and IRGC—with the latter expressed exclusively in the testis. This leaves IRGM as the sole IRG expressed in somatic cells, and unlike its murine counterparts, it is not robustly induced by interferons (54). Instead, IRGM is constitutively expressed at low levels and has been implicated in the regulation of autophagy, including during infections with *M. tuberculosis* and *Salmonella* (55, 56). IRGM has also been associated with Crohn’s disease susceptibility in genome-wide association studies, suggesting a broader role in inflammatory homeostasis (57).

Mouse studies have revealed important insights into IRG function that are difficult to translate directly to humans. For example, Irgm1 deletion in mice worsens disease in Mtb infection, increasing bacterial burden and mortality (58). Yet, the removal of Irgm3 in the same background rescues this phenotype, suggesting complex regulatory interplay among paralogs. This kind of intragenic compensation is unlikely to exist in humans, where only a single somatically expressed IRG gene remains.

These observations raise fundamental questions about the context-specific logic of GTPase-mediated immunity. What functions—distributed across multiple IRGs in mice—are executed by IRGM alone in humans? How is IRGM regulated in the absence of IFN responsiveness? And more broadly, have other gene families in humans (e.g., GBPs, TRIMs, autophagy regulators) taken on functions lost with IRG contraction? These questions speak to a core principle of this review: the architecture of innate immunity is shaped not just by conserved effectors, but by species-specific constraints and compensatory adaptations.

Encouragingly, IRGM’s protein-coding region is intact and amenable to functional manipulation in human cells. This makes it an attractive candidate for allele-specific functional studies, particularly given its polymorphic association with disease and its apparent role in intracellular pathogen restriction. Identifying the molecular partners and upstream signals that govern IRGM’s activation—and determining whether they vary by pathogen or cell type—may clarify how this minimal IRG repertoire is deployed in the human immune system. Such studies could help identify whether IRGM substitutes for multiple effector roles or integrates into alternative GTPase networks altogether.

## CASPASES, INFLAMMASOMES, AND SPECIES-SPECIFIC ARCHITECTURES OF CYTOSOLIC SURVEILLANCE

The role of inflammatory caspases in intracellular pathogen control exemplifies how even core innate immune mechanisms can diverge dramatically across species. In the mouse, caspase-11 (encoded by *Casp4*) functions as a cytosolic sensor of LPS, triggering inflammasome activation, pyroptotic cell death, and IL-1 family cytokine secretion (4, 59, 60). In the Price study, *Casp4* was identified as one of six interferon-stimulated genes (ISGs) required for full IFN-γ-mediated control of *Legionella pneumophila*—a result consistent with prior studies implicating caspase-11 in host defense against Gram-negative intracellular bacteria (2).

In humans, however, the orthologous proteins caspase-4 and caspase-5 show substantial differences in expression patterns, activation thresholds, and functional roles. For example, while both caspase-4 and -5 can respond to cytosolic LPS, caspase-4 appears to be the dominant responder in most primary human macrophages, and its activation is tightly regulated by upstream signals including interferon priming and GBP1 recruitment (61–64). Importantly, human GBPs differ in number, domain structure, and targeting behavior compared to their murine counterparts—affecting how caspase-4 is spatially and temporally activated in the cytosol (7, 65). These observations point to a broader theme: inflammasome architecture is deeply species-specific, not only in gene content but also in regulatory logic, cell-type specificity, and pathogen responsiveness (66–69). Even when effector outcomes appear superficially similar (e.g., IL-1β release), the pathways that generate these outputs differ between species—and often even between macrophage subtypes within a species.

From a context-specific immunity standpoint, the inflammasome is a powerful illustration of how immune logic is shaped by evolutionary, ecological, and physiological constraints. Rather than assuming a conserved inflammasome “blueprint,” we should consider that species (and even cell types within species) may deploy distinct cytosolic sensing programs in response to the same pathogen. In this light, the inclusion of *Casp4* in the IFN-γ-driven control module in mice may not have a direct analog in human macrophages—but the functional role (e.g., responding to LPS+ stress) may be filled by different circuits altogether.

This perspective also reframes a translational challenge as a discovery opportunity: what is the human macrophage equivalent of the caspase-11–GBP–inflammasome axis observed in mice? Are there contextual surrogates—combinations of human-specific GBPs, caspase-4, and priming cues—that function analogously in response to pathogens like *Legionella*, *Salmonella*, or *Shigella*? Mapping these functionally equivalent modules across species will be essential to designing human-specific immunotherapeutic strategies that harness cytosolic sensing without over-relying on murine paradigms.

## PRIMATE-SPECIFIC ANTIMICROBIAL PROGRAMS: DIVERGENCE BY DESIGN

While much of our mechanistic understanding of antimicrobial immunity has been shaped by murine models, an expanding body of work reveals that primates—and humans in particular—express distinct interferon-stimulated genes (ISGs) that either lack clear orthologs in mice or function through unique molecular pathways. These effectors highlight the plasticity of host defense programs and offer compelling examples of functionally equivalent modules that differ in form.

For instance, the interferon-inducible apolipoprotein L3 (APOL3) is expressed in primates but not in rodents and has been shown to restrict replication of cytosolic bacteria such as *Salmonella* Typhimurium by targeting bacterial membranes (70). Similarly, human guanylate-binding proteins (GBPs) differ in number, domain structure, and targeting specificity compared to their murine counterparts. Human GBP1, for example, can directly bind to *Shigella flexneri* in the cytosol and recruit caspase-4, a pathway not observed in mouse cells where caspase-11 and GBPs function differently (71). These differences are not merely structural—they represent alternative execution strategies for cytosolic surveillance.

Other antimicrobial programs show evidence of gene family remodeling in primates. Antimicrobial peptides such as LL-37 and human-specific β-defensins also differ in copy number and inducibility compared to rodents, reflecting divergent selection pressures on epithelial and mucosal defense systems (72–75). The enzyme IDO1, which catabolizes tryptophan and imposes nutritional stress on intracellular pathogens like Toxoplasma gondii and Chlamydia trachomatis, is more robustly induced and functionally deployed in primates than in mice. While murine cells often require non-physiological stimuli to express IDO1, primate cells—especially human macrophages and epithelial cells—readily upregulate IDO1 in response to IFN-γ (49, 76–82). This suggests that primates may have elevated the role of tryptophan starvation as a core antimicrobial strategy, compensating, in part, for the attenuation of other metabolic defenses like nitric oxide or itaconate. Even immunoregulatory proteins such as Siglecs exhibit lineage-specific expansions and polymorphisms in humans that shape macrophage and neutrophil activation thresholds (83).

Together, these examples underscore a central principle of context-specific immunity: that species-specific effectors may evolve not simply as additions to a conserved core, but as replacements or rewiring of entire immune modules. The existence of human- and primate-specific ISGs invites a shift in how we interpret divergence—not as a barrier to translational relevance, but as a roadmap to uncover species-optimized solutions to shared microbial threats.

In this light, mapping the logic of antimicrobial defense requires more than identifying conserved gene names—it requires functional comparisons that account for species-specific expression, regulation, and molecular interactions, with an eye toward how different hosts solve the same biological problem using different immune architectures.

## REWRITING THE LOGIC OF PATHOGEN CONTROL ACROSS SPECIES

The classical approach to studying antimicrobial immunity has often emphasized conserved effector pathways—mechanisms presumed to be broadly shared across mammals and universally applicable to human health. Yet as this review highlights, the molecular logic that governs pathogen control is anything but uniform. The IFN-γ-induced defense programs that control intracellular bacteria in mice are not always replicated in humans, and even when the genes are conserved, their regulation, activation thresholds, and effector outputs may differ in fundamental ways.

Examples such as nitric oxide production, itaconate synthesis, IRG-mediated vacuole disruption, and inflammasome activation all point toward a central conclusion: immune defense strategies are deeply shaped by species-, tissue-, and pathogen-specific context. Murine macrophages rely heavily on NO and itaconate for pathogen restriction, while human macrophages appear to deploy distinct or compensatory pathways, including alternative metabolic programs, inflammasome sensors, and GTPase effectors. Even the seemingly conserved ROS system shows variability in inducibility and deployment, depending on the pathogen niche and immune cell subtype.

This variation should not be viewed as a translational hurdle, but rather as an opportunity to uncover the adaptive tuning of immune architecture. The divergence of IRG gene families or the rewiring of inflammasome modules across species reflects evolutionary solutions to differing microbial ecologies, tissue demands, and lifespan constraints. Within this framework of context-specific immunity, the same pathogen may be cleared via different mechanisms depending on the host species.

What emerges is a vision of innate immunity that is modular, flexible, and evolutionarily plastic. Instead of searching for universal immune blueprints, we should focus on identifying functionally equivalent modules—distinct sets of genes or pathways that fulfill the same protective role in different contexts. Mapping these modules across species and understanding how they are integrated into broader immune networks will be essential for designing next-generation immunotherapeutics that are both potent and contextually appropriate.

Looking ahead, several key priorities emerge.

- Development of enhanced tools for genetic manipulation of primary human phagocytes will facilitate similar studies as those performed by Price and Pilla to define the combinatorial complexity of antimicrobial genes in human cells
- Investigation of naturally occurring human genetic variants in ISGs like *IRG1*, *IRGM*, or *GBP1*, to understand how variation in these genes shapes infection outcomes.
- Development of a comparative immunology toolkit that centers not on conserved markers, but on modularity, redundancy, and network-level behavior of immune pathways.

As we revisit foundational models of intracellular pathogen control through this lens, we move closer to a paradigm that reflects the true diversity of host defense strategies. Embracing context-specific immunity will be essential—not only for translating insights from animal models to humans but also for advancing a more nuanced, evolution-aware approach to immunology.
